# Biodegradable Metals and Corrosion Control: Challenges, Limits and New Opportunities for Innovating in Orthopedic Fixations

**DOI:** 10.3390/ma19091789

**Published:** 2026-04-28

**Authors:** Abdelhakim Cherqaoui, Carlo Paternoster, Diego Mantovani

**Affiliations:** Laboratory for Biomaterials and Bioengineering, Canada Research Chair Tier I, Department of Mineral, Metallurgical, and Materials Engineering, and University Hospital Research Center, Regenerative Medicine, University Laval, Québec, QC G1L 3L5, Canada; abdelhakim.cherqaoui.1@ulaval.ca (A.C.); carlo.paternoster.ulaval1@gmail.com (C.P.)

**Keywords:** fixation implants, biodegradable metals, Fe-Mn-C TWIP alloys, corrosion control, LPBF

## Abstract

Biodegradable metals represent a paradigm shift in orthopedic fixation by providing temporary mechanical support synchronized with bone healing while eliminating long-term complications associated with permanent implants. Conventional bioinert alloys, including stainless steels, Ti-based alloys, and Co-Cr alloys, exhibit high elastic moduli that induce stress shielding and often require secondary removal surgeries. In response, resorbable metallic systems based on Mg, Zn, and Fe have emerged as promising alternatives. Among these, Fe-Mn-C alloys stand out for load-bearing applications due to their exceptional strength-ductility balance governed by twinning-induced plasticity mechanisms, tunable degradation behavior, and intrinsic magnetic resonance imaging compatibility through austenitic phase stabilization. Focusing on Fe-Mn-C alloys, this review critically examines the metallurgical design principles underlying stacking fault energy optimization, phase stability, and Mn-controlled electrochemical behavior. Processing innovations, such as additive manufacturing, are discussed as tools to architecture porosity, refine microstructure, and accelerate degradation by graded designs while preserving mechanical structural support during healing. Hybrid metallic-bioactive systems, surface functionalization strategies, and functionally graded porous architectures were evaluated as advanced approaches to enhance osteointegration and modulate degradability. Despite these advances, significant barriers remain for clinical translation. Persistent discrepancies between in vitro and in vivo degradation rates, often attributed to biological encapsulation and degradation product accumulation, complicate lifetime prediction. Localized corrosion at microstructural heterogeneities such as twin boundaries and phase interfaces can undermine structural reliability under load-bearing conditions. Moreover, predictive multi-physics modeling frameworks capable of coupling electrochemical kinetics, mechanical loading, microstructural evolution, and bone remodeling remain underdeveloped, limiting reliable safety-margin estimation. Regulatory progress is further hindered by the absence of standardized testing protocols specifically tailored to Fe-based biodegradable alloys, including harmonized degradation rate windows, validated corrosion-mechanics coupling methodologies, and clinically defined Mn ion release thresholds. This review aims to discuss whether Fe-based alloys, especially Fe-Mn-C alloys, can transition from promising laboratory materials to clinically viable next-generation orthopedic implants capable of delivering patient-specific, mechanically compatible, and biologically synchronized temporary fixation.

## 1. Introduction

Orthopedic fixation devices play a crucial role in modern trauma care by stabilizing fractured bone segments, maintaining anatomical alignment, and ensuring mechanical support all along the healing process. In the clinical management of complex fractures, internal fixation plates, screws, and intramedullary nails are usually used to provide immediate structural integrity and facilitate biological repair under physiological loading conditions. These devices must withstand significant mechanical forces while minimizing micro-motion at the fracture site to promote effective osseous healing and prevent non-union or malunion [1].

Metallic implants have been widely used in orthopedic surgery for decades due to their superior strength and fatigue resistance compared to polymeric or ceramic alternatives [2]. Commercially established materials such as stainless steel, Ti alloys, and Co-Cr alloys have permit reliable fixation in load-bearing skeletal sites, particularly in long bone fractures that experience substantial mechanical stresses. Their high yield strength and fracture toughness facilitate immediate mechanical stability, which is important for early mobilization and weight-bearing post-procedure [1,2].

However, the ideal fixation device must also harmonize with the body’s dynamic healing environment by providing sufficient support during the early stages of bone regeneration and gradually transferring load back to the healing tissue as biological strength increases. Failure to achieve this balance in load transfer can compromise healing outcomes and impede functional recovery [3].

Commercially established bioinert alloys, including stainless steel 316L, Ti-based alloys (e.g., Ti-6Al-4V), and Co-Cr alloys, have permitted reliable fixation in load-bearing skeletal sites due to their high yield strength, fracture toughness, and corrosion resistance (Table 1). However, their elastic moduli (55–230 GPa) greatly exceed that of cortical bone (10–30 GPa), leading to stress shielding, impaired bone remodeling, and the frequent need for secondary removal surgery [1,2]. In addition, the potential for long-term metallic ion release (Ni^2+^, V, Al, Co) raises biocompatibility concerns under prolonged implantation [1,2]. These inherent limitations, mechanical mismatch, device permanence, and risk of ion toxicity, have motivated growing interest in biodegradable metallic systems capable of providing sufficient short-term load-bearing while progressively resorbing in synchrony with bone healing.

A comparative summary of the mechanical properties, advantages, and limitations of conventional corrosion-resistant metallic biomaterials used for orthopedic fixation is provided in Table 1.

While these metals have demonstrated long-standing clinical success, they were intrinsically designed for permanent implantation, and therefore remain fundamentally incompatible with the concept of temporary orthopedic fixation in a context where tissues are constantly remodeled by cell activity. The pronounced elastic modulus mismatch with bone, the persistence of metallic devices after fracture healing, the potential for long-term ion release, and the frequent need for secondary removal surgeries highlight inherent limitations when such bioinert alloys are used in applications requiring only transient mechanical support. These shortcomings have driven growing interest in biodegradable metallic systems capable of providing sufficient short-term load-bearing while progressively degrading and eliminating long-term complications.

The limitations of permanent bioinert metals have motivated the development of biodegradable metallic implants that can provide temporary mechanical support and subsequently resorb in vivo, eliminating the need for surgical removal and mitigating long-term complications. Biodegradable metals (BMs) are designed to gradually degrade in physiological environments, ideally in synchrony with the biological timeline of tissue healing. Their degradation products must be non-toxic and safely metabolized or excreted by the body [8,9].

Among candidate BMs, Mg, Zn, and Fe have attracted considerable attention due to their mechanical performance, biodegradation behavior, and biological compatibility. Mg alloys exhibit an elastic modulus closer to native bone and can stimulate osteogenic activity, but their rapid corrosion and hydrogen gas evolution are significant challenges. Zn alloys demonstrate intermediate degradation rates (DRs) and biological functionality but often lack mechanical strength for heavy load-bearing applications. Finally, Fe-based systems offer superior mechanical integrity but suffer from slow degradation [10].

The core design challenge for BMs lies in balancing mechanical integrity, DRs, and biological response, ensuring that implanted devices maintain structural function during healing while degrading at a rate that avoids adverse reactions and aligns with tissue regeneration [9].

This review critically examines the development and the performance of BMs for orthopedic fixation, with particular emphasis on Fe-based biodegradable alloys, which offer superior load-bearing capability. In this regard, we analyze alloy design strategies, degradation behavior, and biological responses in physiological environments. Within this framework, Fe-Mn-C alloys are highlighted as promising candidates due to their potential for austenitic stabilization, reduced magnetic susceptibility, favorable mechanical properties, and enhanced corrosion control.

The influence of processing methods, including powder metallurgy (PM) and laser powder bed fusion (LPBF), on porosity, microstructure, and degradation behavior is examined. We also explore correlations between alloy composition, structure-property relationships, and DRs, with a focus on tailoring BMs for temporary orthopedic fixation.

Finally, this review identifies current challenges and limitations toward clinical translation and outlines new opportunities for innovation in next-generation of biodegradable metallic implants capable of providing temporary mechanical support while safely resorbing following bone healing.

## 2. Mechanical, Degradation, and Biological Requirements for Biodegradable Orthopedic Implants

The design and development of BMs for orthopedic fixation require the integration of mechanical performance, degradation behavior, and biological safety. Unlike permanent implants, biodegradable devices must retain sufficient structural integrity during the critical healing phase and subsequently degrade in a controlled and biologically acceptable manner. Therefore, clearly defined performance criteria are necessary to evaluate candidate materials and guide alloy design strategies [11,12,13].

### 2.1. Mechanical Compatibility

Mechanical compatibility with bone is a fundamental requirement for orthopedic implants. Cortical bone exhibits an elastic modulus typically ranging from 10 to 30 GPa, while trabecular bone presents lower values between 0.1 and 5 GPa depending on anatomical location and density [14]. In contrast, conventional metallic biomaterials exhibit significantly higher stiffness, resulting in mechanical mismatch and stress shielding, which reduces physiological load transfer, and may impair bone remodeling [3,15].

**Figure 1:**
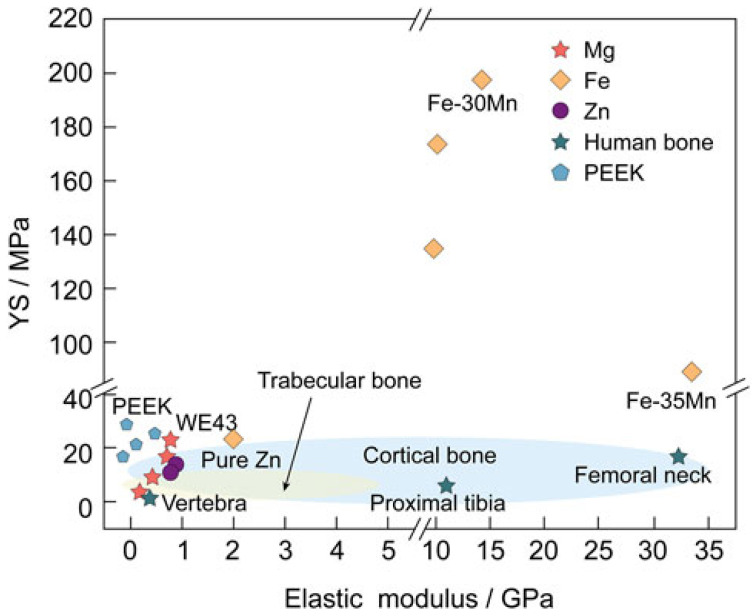
Elastic modulus (GPa) and yield strength (YS/MPa) comparison of cortical bone, trabecular bone, PEEK, and biodegradable metallic systems. Reprinted from Wang et al. (2022) [17], licensed under CC BY 4.0.

As depicted in Figure 1, the reduction in implant stiffness toward values closer to cortical bone is desirable to promote load sharing and preserve mechanical stimulation of surrounding tissue. However, elastic modulus alone cannot define mechanical compatibility. Biodegradable fixation devices must also provide sufficient yield strength (YS) and fatigue resistance to withstand physiological stresses throughout the healing period [11,16]. Cortical bone exhibits compressive strengths (CP) between 100 and 200 MPa, and implants must demonstrate comparable or superior YS to ensure mechanical stability [14,15]. In long bone fracture fixation, cyclic loading induced by walking and daily activities makes fatigue resistance a critical parameter.

Fatigue resistance under cyclic physiological loading constitutes an additional critical mechanical requirement that is often underweighted in biodegradable metal design. During normal ambulation, bone fixation devices are subjected to repetitive loading cycles in the range of 10^5^–10^7^ cycles over the healing period, with peak stresses that can reach 50–150 MPa depending on anatomical location and patient activity level. Load-bearing biodegradable implants must therefore demonstrate fatigue limits exceeding 100 MPa to ensure structural integrity throughout the healing timeline (Table 2).

Importantly, mechanical integrity must be preserved during the degradation process. Premature loss of strength before adequate bone regeneration may lead to implant failure or delayed union, highlighting that mechanical performance and DR are intrinsically coupled variables in BMs design [12,18]. Furthermore, mechanical requirements vary depending on anatomical location and clinical indication. In this regards, non-load-bearing implants may tolerate lower strength and faster degradation, whereas load-bearing applications demand high initial strength and gradual, predictable loss of mechanical integrity.

**Table 2:** Mechanical requirements for biodegradable metallic implants in orthopedic applications (load-bearing and non-load-bearing) [13,19,20,21].

	E (GPa)	YS (MPa)	UTS (MPa)	Fatigue Limit (MPa)	A (%)	DR (Months)	Strength Retention (%) After 3 Months
Cortical bone (Benchmark)	10–30	100–200	100–300	50–100	~2–5 (bone)	-	-
Load-bearing implant target	~20–80	≥200	≥300	>100	>10	3–6	≥50%
Non-load-bearing implant target	~10–40	≥100	≥150	>70	>5	1–3	≥30%

Within this framework, Fe-based systems are particularly attractive for load-bearing scenarios due to their superior mechanical properties compared with Mg- and Zn-based alloys [13,22]. Nevertheless, achieving optimal mechanical compatibility is insufficient on its own. Here, long-term success critically depends on how the material degrades in vivo.

### 2.2. Degradation Requirements

Once adequate mechanical stability is ensured, degradation behavior becomes the determining factor for sustained implant performance. Ideally, a biodegradable bone contact implant should degrade in synchrony with bone healing, which typically occurs over 3 to 6 months for uncomplicated fractures, although this period may vary according to patient-specific and anatomical factors [23]. Excessively rapid degradation compromises mechanical support, whereas overly slow degradation undermines the fundamental clinical advantage of biodegradability.

**Figure 2:**
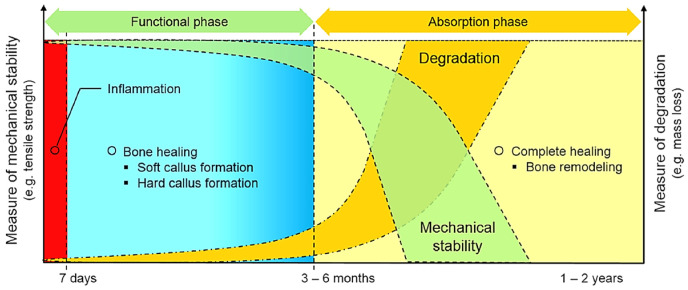
Schematic representation of the temporal relationship between bone healing stages and the required degradation profile of a biodegradable metallic implant. The implant must maintain sufficient mechanical integrity through the inflammatory (1–7 days) and reconstruction (3–6 months) phases, while progressively degrading to allow complete resorption by the bone remodeling stage (1–2 years). Reproduced from Sarian et al. [24], under CC-BY 4.0 license.

As conceptually illustrated in Figure 2, the optimal degradation profile involves gradual reduction in mechanical integrity that parallels the increasing load-bearing capacity of healing bone. Achieving this balance remains one of the main challenges in biodegradable alloy development. Mg alloys often exhibit excessively high DR in physiological environments, whereas pure Fe degrades too slowly, necessitating alloying strategies and microstructural modifications to tailor degradation kinetics [11,22]. In this regards, Table 3 summary representative studies confirming this trend in DR, along with their corresponding experimental conditions.

Beyond the overall DR of the implant, the mode of degradation critically affects structural reliability. Uniform degradation allows predictable and homogeneous reduction in cross-sectional area, while localized corrosion phenomena such as pitting generate stress concentration sites that may trigger premature mechanical failure [12].

Microstructural characteristics including grain size, secondary phases distribution, porosity, and processing-induced defects strongly influence corrosion morphology. Additive manufacturing (AM) routes, for instance, may introduce residual stresses and microstructural heterogeneities that locally modify electrochemical behavior [25]. In addition, degradation products must remain physiologically manageable. For example, hydrogen evolution represents a major concern in Mg-based systems, as rapid degradation may produce gas cavities that interfere with tissue integration [11,26]. On the other hand, although Fe- and Zn-based alloys do not generate significant hydrogen, their degradation products must be gradually resorbed without long-term accumulation [13,22].

**Table 3:** Example of studies showing the typical DRs characteristics of BMs.

Biodegradable Metal System	Typical DR (mmpy)	Experimental Context	Ref.
Mg-based alloys (e.g., WE43, AZ31, etc.)	~0.5–4 mmpy (often >1 mmpy)	In vitro corrosion tests in simulated body fluid accompanied with rapid hydrogen evolution	[27]
Zn-based alloys (e.g., Zn-Mg, Zn-1 wt.% alloy)	~0.1–0.3 mmpy	In vitro electrochemical/immersion studies of Zn alloys showing moderate corrosion	[28]
Zn (pure or binary)	~0.17–0.22 mmpy	Immersion tests in simulated body fluids with biological alloying elements	[29]
Fe-based scaffolds (dense pure Fe)	~0.03–0.09 mmpy	In vitro immersion tests showing slower degradation for Fe scaffolds	[28]
Fe-based (Fe25Mn scaffolds)	~0.23 mmpy	In vitro tests showing accelerated corrosion in Mn-containing Fe alloys	

Because degradation directly governs ion release and local chemical changes, degradation behavior cannot be separated from biological response. This interdependence naturally leads to the final essential requirement, which is the biological compatibility.

### 2.3. Biological Performances

The biological response to biodegradable metallic implants is fundamentally linked to ion release, degradation products, and the local microenvironment generated during degradation. As degradation progresses, metallic ions such as Fe^2+^, Mg^2+^, and Zn^2+^ are released into surrounding tissues. While these elements are essential trace elements involved in metabolic and enzymatic pathways, excessive local concentrations may induce cytotoxicity, oxidative stress, or inflammatory reactions if degradation is not properly controlled [11,13]. Table 4 summarizes the biological roles of these ions, typical physiological concentration ranges, and potential toxicological consequences if local or systemic levels become elevated due to implant degradation.

Therefore, DRs must ensure that ion concentrations remain below toxicological thresholds while still permitting gradual material resorption. In Fe-based alloys, slower degradation may reduce acute toxicity but may also prolong the presence of degradation products in vivo, emphasizing the need for balanced degradation control strategies [22]. Beyond ion release, successful implant integration requires support of osteoblast adhesion and proliferation, alongside minimization of chronic inflammatory responses. Macrophage interactions with degradation products play a central role in regulating tissue remodeling and implant resorption [12,26]. Although in vitro assays provide preliminary assessment of cytocompatibility, in vivo environment introduces complex interactions involving proteins, immune cells, vascularization, and mechanical loading that dynamically influence degradation behavior [18].

Finally, imaging compatibility has emerged as an additional clinical consideration. MRI is frequently employed for post-operative monitoring of fracture healing. In this context, materials exhibiting strong ferromagnetic behavior may generate imaging artifacts that compromise diagnostic accuracy. Austenitic Fe-Mn alloys, characterized by reduced magnetic susceptibility compared with pure Fe, have therefore been proposed as promising candidates for MRI-compatible Fe-based biodegradable alloys [32]. This concept of imaging compatibility will be detailed in the upcoming sections of this review.

## 3. Biodegradable Metals and Alloys for Orthopedic Fixations: State of the Art

BMs investigated for orthopedic fixation are primarily based on Mg, Zn, and Fe. Each alloy system presents a distinct balance between mechanical performance, degradation, and biological response. While Mg alloys were the first extensively explored class, Zn- and Fe-based alloys have emerged as alternatives to overcome specific mechanical or degradation limitations. The following sections critically examine the advantages and challenges associated with each system.

### 3.1. Mg-Based Alloys

Mg and its alloys were among the earliest metallic systems proposed for biodegradable orthopedic applications. Their primary advantage lies in their density (1.74 g/cm^3^) and elastic modulus (41–45 GPa), which are closer to those of cortical bone compared with conventional metals used for permanent implants. This similarity reduces stress shielding and promotes physiological load transfer [33]. In addition, Mg is biocompatible and participates in natural metabolic pathways, serving as a cofactor in several enzymatic reactions.

Clinically, Mg-based devices such as screws and pins have reached commercialization, notably Mg-Y-RE-Zr (MAGNEZIX^®^) with CE approval and Mg-Zn-Ca systems (RESOMET^®^) approved by KFDA [34]. These regulatory approvals represent a major milestone in biodegradable metallic implants.

Recent progress in Mg-based alloy development has focused on two complementary fronts, alloy chemistry optimization and surface engineering. On the alloying side, rare-earth-free systems such as Mg-Zn-Mn and Mg-Ca-Sr have demonstrated promising combinations of moderate DRs (0.3–0.8 mmpy) and improved mechanical stability without the concerns associated with rare-earth element toxicity and regulatory status [34]. Thermomechanical processing, including equal channel angular pressing (ECAP) and extrusion, has enabled grain refinement to sub-5 µm levels, improving both strength and ductility while reducing corrosion anisotropy [34].

Coating strategies have evolved considerably, with plasma electrolytic oxidation (PEO) and composite polymer-ceramic coatings providing initial protection during the critical early healing phase while permitting progressive dissolution thereafter [34].

However, despite these advantages, Mg alloys suffer from intrinsic limitations. Mechanically, although tensile strengths (TS) between 135–285 MPa can be achieved, ductility and fatigue resistance remain concerns for high-load applications. More critically, Mg exhibits rapid and often uncontrolled degradation in physiological environments, with in vitro DRs frequently exceeding 1 mmpy. This rapid degradation generates Mg^2+^ ions and hydrogen gas through cathodic reactions, potentially leading to subcutaneous gas pockets, osteolysis, and premature mechanical failure [12,33,34].

Therefore, the main challenge in Mg-based systems remains the control of DRs through alloying (e.g., Zn, Ca, rare earth elements), thermomechanical processing, and surface modification strategies. While significant progress has been achieved, rapid degradation still restricts Mg alloys primarily to low- to moderate-load bearing applications.

### 3.2. Zn-Based Alloys

Zn-based alloys have emerged as an intermediate solution between rapidly degrading Mg and slow degradation of Fe. Zn exhibits a moderate DR in physiological conditions, typically ranging between 0.02–0.3 mmpy, which aligns more closely with the bone healing timeline. Importantly, Zn does not produce hydrogen gas during degradation, thereby eliminating the risk of gas cavity formation [35,36].

From a biological point of view, Zn is an essential trace element involved in bone metabolism and osteoblast proliferation [37,38]. This intrinsic bioactivity makes Zn particularly attractive for orthopedic applications. However, pure Zn suffers from low mechanical strength (YS: 20–50 MPa), limiting its direct use in load-bearing fixation devices. To overcome this limitation, extensive alloying strategies have been developed. Zn-Mg, Zn-Li, Zn-Ca, and Zn-Ag systems have demonstrated significant improvements in strength, with ultimate tensile strengths (UTS) reaching 360–560 MPa in Zn-Li alloys [39,40]. Alloying refines microstructure, enhances precipitation strengthening, and enables tuning of degradation behavior.

Recent studies have demonstrated that thermomechanical processing, including hot extrusion and rolling with controlled reduction ratios, can markedly improve the mechanical performance of Zn-based alloys beyond what is achievable through com-position alone. Extrusion-induced texture and grain refinement in Zn-Mg and Zn-Li al-loys have yielded UTS values approaching 400–560 MPa with elongations exceeding 15–20%, making these systems increasingly competitive for low- to moderate-load-bearing applications [39,40].

Importantly, creep deformation under sustained physiological loading represents a recognized but insufficiently addressed challenge for Zn-based alloys. Given the relatively low melting point of Zn (419 °C), the homologous temperature of a Zn implant at body temperature (37 °C) is approximately 0.68 Tm, a range well within the regime where thermally activated creep mechanisms, particularly grain boundary sliding and dislocation climb, become operative under sustained physiological loads [41]. This susceptibility to dimensional instability caused by creep over several months of in vivo loading may compromise the effectiveness of the fixation and requires specific mechanical characterization within the context of clinically relevant loading protocols.

Altogether, Zn-based alloys represent a promising compromise in terms of DR and biological compatibility, yet their mechanical performance still requires further enhancement for widespread orthopedic fixation use.

### 3.3. Fe-Based Alloys

Fe-based biodegradable alloys are distinguished by their superior mechanical strength. Pure Fe exhibits YS around 170 MPa and UTS between 200–270 MPa, while Fe-Mn alloys can achieve UTS values of 400–900 MPa with enhanced ductility [42,43]. These values are comparable to conventional structural metals, making Fe-based alloys particularly attractive for load-bearing orthopedic applications.

However, the high elastic modulus of Fe (~210 GPa) significantly exceeds that of cortical bone, raising concerns about stress shielding [41]. Strategies to reduce effective stiffness include alloying with Mn to stabilize the austenitic phase, introducing porosity via PM and AM, and microstructural engineering to tailor mechanical response.

Additionally, the principal limitation of Fe-based alloys as BMs is their slow DR in physiological environments. Dense pure Fe typically exhibits DRs around 0.1 mmpy, which may be insufficient for complete resorption within clinically relevant timeframes. To accelerate degradation, several strategies have been explored:

- Alloying additions (Mn, Pd, Ag, C) to destabilize passive films;
- Galvanic coupling using second-phase particles;
- Porosity engineering to increase surface area;
- Grain refinement to enhance electrochemical activity.

Biologically, Fe is essential for oxygen transport and cellular metabolism. Controlled ion release generally demonstrates good cytocompatibility, even though excessive accumulation may induce oxidative stress through reactive oxygen species (ROS) generation [44,45]. Therefore, tailoring DR without compromising mechanical performances remains the main research challenge for Fe-based biodegradable alloys.

Given their superior mechanical properties and tunable degradation behavior, Fe-based alloys are increasingly considered strong candidates for load-bearing biodegradable fixation implants. Among the various Fe-based alloys investigated, Fe-Mn-based have attracted particular attention due to their ability to stabilize the austenitic phase, reduce magnetic susceptibility, and enhance degradability compared to pure Fe. However, binary Fe-Mn alloys may still suffer from limited phase stability and strength-ductility balance depending on Mn content. The addition of C introduces an additional degree of metallurgical control by further stabilizing austenite, increasing stacking fault energy (SFE), and enabling twinning-induced plasticity (TWIP) mechanisms. Consequently, Fe-Mn-C alloys emerge as a highly promising class of BMs, offering a unique combination of mechanical performance, tunable degradation behavior, and MRI compatibility [46,47,48,49]. Their metallurgical design principles are discussed in the following section.

To synthesize the current state of the art, Table 5 provides a comparative overview of the three principal biodegradable metallic systems.

## 4. Metallurgical Design of Fe-Mn-C Biodegradable Alloys

Fe-Mn-C alloys represent a highly tunable class of Fe-based biodegradable materials in which phase stability, mechanical behavior, magnetic response, degradation, and biological performance can be simultaneously engineered. Their design requires an integrated metallurgical strategy that balances austenitic stabilization, SFE optimization, electrochemical activation, and processing control to ensure reliable in vitro and in vivo performance.

### 4.1. Austenitic Stabilization Mechanisms

For biodegradable orthopedic implants requiring post-operative MRI monitoring, magnetic behavior constitutes a critical constraint. Pure Fe is ferromagnetic and exhibits high magnetic susceptibility (~1.5 × 10^6^) [56], which severely disturbs the homogeneity of the magnetic field during MRI scanning, leading to signal loss, geometric distortions, and RF-induced heating risks [57,58,59,60]. Ferromagnetic stainless steels have been reported to generate pronounced “black-hole” artifacts and image distortion, significantly compromising diagnostic quality [58]. Consequently, stabilization of non-ferromagnetic phases is essential in Fe-based biodegradable alloys.

Alloying Fe with Mn promotes stabilization of γ-austenite (FCC) and ε-martensite (HCP), both of which are antiferromagnetic or weakly paramagnetic, thereby substantially reducing magnetic susceptibility and improving MRI compatibility [61]. However, formation of α′-martensite must be strictly avoided due to its ferromagnetic nature. For instance, Filho et al. [62] reported that the magnetic susceptibility of an Fe-17.6Mn alloy increased as the fraction of α’-martensite increased. From a mechanical standpoint, a fully γ-austenitic structure is preferred due to its superior ductility and magnetic neutrality, although a γ + ε dual-phase microstructure may remain acceptable in the condition that ε-martensite fraction is limited and α′ is suppressed [61].

C plays a decisive role in this stabilization strategy. As a strong interstitial γ-stabilizer, C suppresses martensitic transformation, enhances solid-solution strength-ening, and contributes to SFE control. Nevertheless, C has the tendency to form car-bide precipitates at elevated temperature, which must be carefully managed. Within the temperature range of approximately 500–700 °C, C preferentially segregates to grain boundaries and precipitates as cementite (Fe_3_C) or the ternary (Fe,Mn)_3_C phase [57,58,59,60]. This process depletes adjacent regions of both C and Mn, locally reducing austenite stability and SFE, and creating compositionally heterogeneous zones that may act as preferential anodic sites in physiological environments.

The resulting sensitization phenomenon is analogous to intergranular corrosion sensitization in austenitic stainless steels caused by Cr carbide precipitation at grain boundaries, where the Cr-depleted zones adjacent to carbides become electrochemically active and susceptible to preferential dissolution in corrosive media [61]. In Fe-Mn-C alloys, sensitization could similarly promote preferential grain boundary attack and undermine the material’s corrosion performance in physiological fluids. To mitigate this risk, solution annealing at temperatures above 1000 °C followed by rapid water quenching is recommended to maintain C and Mn in solid solution and ensure microstructural homogeneity before final processing [57,58,59,60].

Based on these compositional constraints, target compositions for Fe-Mn-C biodegradable TWIP alloys are generally situated in the range of 15–20 wt.% Mn and 0.6–0.8 wt.% C, which concurrently ensures full γ-austenite stabilization, α′-martensite suppression, and SFE values conducive to twinning-dominated plasticity [63].

### 4.2. Stacking Fault Energy and TWIP Effect

Beyond phase stability, the mechanical performance of Fe-Mn-C biodegradable alloys is intrinsically linked to their deformation mechanisms, which are predominantly governed by the SFE. When the SFE falls within the range of 12–35 mJ/m^2^, mechanical twinning becomes the dominant deformation mode, leading to the twinning-induced plasticity (TWIP) effect. This effect enhances both strength and ductility by introducing coherent twin boundaries that impede dislocation motion and promote strain hardening, which is particularly beneficial for load-bearing orthopedic applications [64]. This deformation behavior has been extensively reported in casted Fe-Mn-C TWIP steels. For instance, Loffredo et al. [65] developed a casted Fe-16Mn-0.7C that exhibited a SFE in the range of 18–25 mJ/m^2^, favoring twinning as the main deformation mode, which led to an A = 54.7 ± 2.1%, and UTS = 1946 ± 11 MPa. Also, Dumay et al. [66] demonstrated that Fe-22Mn-0.6C steels exhibit exceptional A (>50%) and UTS (>900 MPa) due to mechanical twinning at a SFE of approximately 20 mJ/m^2^. To achieve such desirable mechanical properties, precise compositional control is essential. Both Mn and C increase the SFE, with C having a more pronounced effect. Utilizing tools like the SFE map and the Schumann diagram [63]. permit the identification of optimal composition ranges that stabilize the desired austenitic phase and maintain the SFE within the target range. For instance, a composition of Fe-16Mn-0.7C falls within this optimal window, promoting mechanical twinning while avoiding the formation of ε-martensite and α′-martensite (associated with SFE <12 mJ/m^2^).

### 4.3. Elastic Modulus

Although Fe-Mn-C alloys can achieve outstanding strength and ductility, their intrinsic elastic modulus (~210 GPa) remains substantially higher than that of cortical bone (10–30 GPa), raising concerns regarding stress shielding. Alloying with Mn slightly reduces stiffness by stabilizing the γ-phase, but the effect is limited. Consequently, microstructural refinement and structural design become essential strategies for tailoring effective modulus without sacrificing strength.

Grain refinement and twinning-induced strain hardening enhance strength while maintaining stable deformation behavior. More significantly, PM and AM enable controlled porosity, which reduces effective elastic modulus and simultaneously increases surface area, thereby accelerating degradation. For instance, porous Fe-Mn alloys with 42–76% porosity exhibited DRs up to 2.71 mmpy, significantly higher than dense counterparts [67], while Heiden et al. [68] demonstrated that salt-leached porous Fe-Mn composites increased DR from 0.02 to 0.82 mmpy and enhanced apatite formation.

The influence of porosity on mechanical properties and degradation rate is summarized in Table 6.

While the degradation-accelerating benefits of porosity are well established, it is critical to consider the concurrent and significant adverse effects on mechanical performance. As systematically shown in Table 6, increasing porosity from below 5% in dense Fe-Mn alloys to 70–85% in highly porous scaffolds reduces compressive strength from above 400 MPa to values of 6–50 MPa, well below the ≥200 MPa threshold required for load-bearing implant applications (Table 2). This inverse relationship between porosity and strength is well described by the Gibson-Ashby cellular solid scaling law, which predicts near-exponential strength reduction with increasing void fraction [68].

Moreover, fatigue performance is affected by porosity. Here, stress concentration factors at strut-junction intersections under cyclic loading greatly amplify the effective stress experienced by the material, reducing fatigue life by orders of magnitude relative to dense counterparts [68]. This mechanical-degradation link constitutes a design paradox for Fe-based biodegradable fixation, as the porosity levels required to achieve clinically acceptable DRs (>0.5 mmpy) simultaneously compromise the structural integrity necessary for sustained load transfer during bone healing. Functionally graded architectures, discussed in Section 7.1, represent a promising but as yet insufficiently validated approach to mitigate this conflict by spatially distributing dense and porous regions according to local mechanical and degradation requirements.

## 5. Processing and Their Influence on Structure–Property–Degradation Relationships

The DRs and mechanical performances of Fe-based biodegradable alloys are not determined solely by composition, but strongly governed by processing-induced microstructure. Processing routes define porosity levels and distribution, phase constitution (γ-austenite vs. ε-martensite), grain size, and defect formation, which collectively determine electrochemical heterogeneity and DRs in physiological environments. Increasing evidence shows that manufacturing strategy acts as a primary design variable for accelerating degradation in Fe-based alloys that are otherwise intrinsically slow-degrading in physiological environments [22,67].

### 5.1. Powder Metallurgy

PM is widely employed for Fe-based biodegradable alloys due to its flexibility in composition control and its inherent ability to introduce engineered porosity. During sintering, diffusion bonding and partial densification determine residual pore fraction and Mn homogenization. In this regard, incomplete Mn diffusion may result in local phase heterogeneity, promoting electrochemical differences between regions and influencing corrosion behavior towards more localized mechanism [70].

A major advantage of PM processing is the ability to tailor porosity as a degradation accelerator. Interconnected pores increase surface area and allow deep electrolyte penetration, enhancing anodic dissolution. Heiden et al. [68] demonstrated that increasing porosity in porous Fe significantly increased DRs while simultaneously reducing elastic modulus toward bone-like values. Similarly, Li et al. [54] reported that additively manufactured porous Fe structures exhibited accelerated corrosion compared to dense Fe due to increased internal surface area and electrolyte accessibility.

Moreover, Fe-Mn alloys produced by PM exhibit DRs significantly higher than pure Fe, primarily due to Mn-induced phase stabilization and modified electrochemical behavior [70]. The combined influence of porosity and alloying creates a processing-structure-degradation coupling that can increase DRs from ~0.1 mmpy in dense Fe to values approaching or exceeding 1 mmpy in highly porous systems [54,68]. These relationships are synthesized in Table 7, which highlights the structural mechanisms by which PM processing accelerates degradation.

### 5.2. Laser Powder Bed Fusion

LPBF modifies the structure–processing–degradation relationship in Fe-Mn and Fe-Mn-C biodegradable alloys through rapid solidification and process-induced microstructural features. Unlike PM, where porosity primarily governs degradation, LPBF influences biodegradation via phase evolution, defect formation, element distribution, and grain refinement, each of which can alter electrochemical activity [28,71].

One of the first systematic investigations of Fe-Mn alloys produced via LPBF found that scanning speed strongly affects microstructure and corrosion behavior. Here, increasing scanning speed increased porosity from ~0.27% to ~2.5% in Fe-Mn alloys processed at scanning speeds between 600–900 mm/s, and resulted in mixed phase structures composed of ε-martensite and α′-martensite. Electrochemical testing in SBF showed that corrosion rates (CRs) ranged from ~0.09 mmpy at low scanning speed to ~0.22 mmpy at intermediate speeds, which were higher than CRs of pure Fe and many conventionally manufactured Fe-Mn alloys [71]. This demonstrates that LPBF process parameters alone can be used to tailor corrosion behavior via microstructural control.

Importantly, the phase composition produced by LPBF can dominate degradation behavior. A recent study directly comparing LPBF-fabricated pure Fe with Fe-25Mn and Fe-30Mn alloys found that adding Mn did not always increase CR when processed by LPBF. The rapid cooling inherent to LPBF produced a refined grain structure in pure Fe with high grain boundary density, resulting in a CR of ~0.04 mmpy. Fe-25Mn showed a similar CR (~0.05 mmpy) due to a high ε-martensite to γ-austenite ratio promoting microgalvanic corrosion. In contrast, Fe-30Mn displayed a lower CR (~0.01 mmpy) associated with coarse columnar grains and reduced microgalvanic effects [72]. These findings underscore the complex interplay between phase distribution and corrosion behavior in Fe-Mn alloys produced via LPBF.

Beyond Fe-Mn binaries, broader reviews indicate that LPBF enhances corrosion in Fe alloys relative to conventional manufacturing due to hierarchical porosity, residual stresses, and destabilized passive films caused by rapid cooling and high defect density. For instance, in LPBF Fe scaffolds, regions near the surface showed faster degradation due to initial attack, while the scaffold core remained relatively intact over short immersion durations, highlighting localized corrosion behavior shaped by LPBF microstructure [28].

Taken together, these studies illustrate that degradation in LPBF Fe-Mn and related alloys is controlled not just by porosity but by solidification-induced microstructural features, such as grain size, phase mixture, and defect distribution. The primary LPBF processing-structure-degradation links are presented in Table 8.

These LPBF studies confirm that even in the absence of designed porosity, corrosion can be significantly influenced by microstructure, which is a direct consequence of rapid thermal cycling and solidification during LPBF. As a result, processing parameters provide a potent control lever for biodegradation in Fe-Mn and Fe-Mn-C alloys, complementing traditional alloy design strategies.

Finally, it’s worth mentioning here that the LPBF process generates extreme thermal gradients and cooling rates up to 10^8^ K/s, producing a spatially graded microstructure that directly governs mechanical performance and degradation behavior [71,72]. The ratio of temperature gradient (G) to solidification rate (R) controls grain morphology across the build direction. Here, high G/R promotes columnar grains while low G/R favors equiaxed structures, introducing anisotropy in both mechanical and corrosion responses [71,72]. In austenitic stainless steels, Cr/Mo microsegregation along cellular boundaries creates preferential sites for pitting and intergranular corrosion, while retained austenite and martensite laths in martensitic steels affect hardness and stress corrosion cracking resistance [71,72]. Superimposed residual stresses from cyclic thermal loading further amplify degradation under fatigue or corrosive environments. Grain-refining additives (e.g., TiC, Y_2_O_3_) and computational modeling approaches are promising tools to optimize these gradient structures toward targeted property-degradation profiles [73].

## 6. Current Challenges and Limitations Towards Clinical Translation

Despite the remarkable progress achieved in Fe-based biodegradable alloys, particularly Fe-Mn and Fe-Mn-C TWIP steels, their clinical translation remains constrained by several unresolved scientific and technological challenges. These include insufficient control of DRs, localized corrosion leading to premature mechanical failure, limited predictive modeling capabilities, scarcity of long-term in vivo validation, and the absence of standardized evaluation protocols specific to Fe-based biodegradable systems.

### 6.1. Limited Control of Degradation and In Vitro-In Vivo Discrepancy

One of the most persistent limitations of Fe-based biomaterials is their intrinsically slow DR under physiological conditions. Pure Fe typically degrades at rates below 0.1 mmpy in simulated body fluids, which is insufficient for many orthopedic applications requiring complete resorption within 1 to 2 years [74,75]. Although alloying with Mn has been shown to increase degradation by lowering the corrosion potential and destabilizing passive films, the acceleration remains moderate in dense systems [76,77]. PM approaches introducing high porosity (e.g., 80–85%) have achieved DRs approaching 0.8–1.0 mmpy in vitro [78], while galvanic strategies using noble elements such as Pd further increase CR [79]. However, in vivo investigations consistently demonstrate slower degradation compared to in vitro immersion tests due to the accumulation of phosphate- and carbonate-rich degradation product layers that act as diffusion barriers [80,81]. For example, long-term implantation studies in rat femora revealed persistent multilayer degradation products even after 52 weeks, significantly retarding further dissolution [77]. This discrepancy between laboratory testing and biological reality highlights a major translational gap. Table 9 summarizes some examples about the divergence between in vitro and in vivo degradation of Fe-based biodegradable alloys.

### 6.2. Localized Degradation and Degradation–Mechanical Integrity Coupling

Beyond the slow degradability of Fe-based biodegradable alloys, the mode of degradation represents a critical mechanical concern. Uniform degradation is desirable for gradual load transfer during bone healing, yet Fe-Mn-C systems frequently exhibit microstructurally driven localized attack. Grain boundary corrosion has been observed in cast Fe-Mn-C TWIP alloys during early immersion stages [86], while multiphase systems containing ε-martensite or noble alloying additions may develop microgalvanic coupling between phases [79]. For example, in Fe-Mn-Ag alloys, martensitic regions promoted localized pitting rather than homogeneous dissolution [87]. Such localized corrosion can generate stress concentration sites and accelerate mechanical integrity loss under load-bearing conditions.

In this regard, Fe-Mn-C alloys derive their exceptional ductility and strength from dynamic mechanical twinning, where twin boundaries act as effective barriers to dislocation motion and sustain high strain-hardening rates [47,88]. However, several studies suggest that these crystallographic features may also act as electrochemically active sites under corrosive conditions. For instance, Loffredo et al. [86] reported preferential corrosion initiation along grain boundaries in cast Fe-Mn-C TWIP steels during early immersion in simulated physiological solutions, indicating that microstructural heterogeneities influence corrosion localization. Furthermore, Kraus et al. [77] observed selective attack at microstructural interfaces in Fe-Mn-based alloys during long-term in vivo implantation, particularly in alloys containing martensitic regions or phase heterogeneities.

From a metallurgical perspective, twin boundaries represent regions of altered atomic stacking and local strain fields, which may modify local electrochemical potential and passive film stability [47]. Specifically, the passive oxide film at twin interfaces, predominantly composed of MnO, Fe_2_O_3_, and Fe_3_O_4_, may be destabilized by the elevated dislocation density and altered Mn distribution resulting from short-range diffusion during deformation [47]. Similar behavior has been reported in austenitic steels, where deformation-induced defects and phase interfaces act as preferential corrosion sites due to localized energy variations and galvanic coupling effects [73]. It should be noted that the susceptibility of twinning boundaries to corrosion is proportional to prior mechanical deformation, since a higher density of twins increases the total length of the susceptible boundary area exposed to physiological electrolyte [47]. In multiphase Fe-Mn systems, microgalvanic interactions between γ-austenite and ε-martensite have also been proposed to accelerate localized dissolution [89].

**Figure 3:**
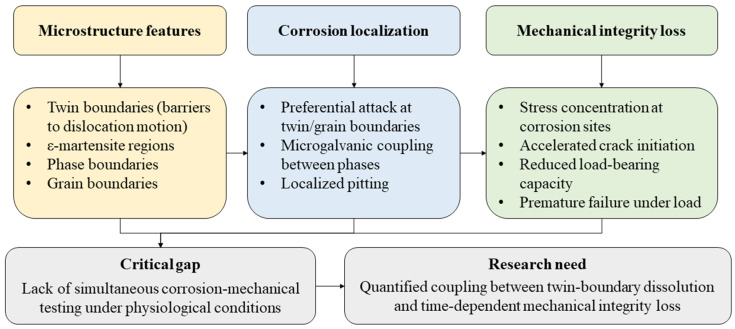
Interaction between microstructure, corrosion localization, and mechanical integrity loss in Fe-Mn-C alloys.

While these studies indicate that microstructural features governing mechanical performance can simultaneously influence corrosion pathways, the direct coupling between twin-boundary dissolution and time-dependent mechanical integrity loss remains insufficiently quantified. Loss of twin boundary coherency through progressive corrosive attack could reduce the effectiveness of dislocation barriers and shift deformation behavior from TWIP-dominated plasticity toward more conventional slip mechanisms [89], with direct implications for the strength-ductility balance required for load-bearing fixation. Current degradation studies typically evaluate mass loss or electrochemical parameters without simultaneous mechanical testing under physiological conditions. Consequently, the relationship between corrosion morphology evolution and load-bearing capacity reduction in Fe-Mn-C biodegradable alloys remains poorly understood. A conceptual framework describing this corrosion–mechanical coupling is therefore proposed in Figure 3.

### 6.3. Limitations of Predictive Modeling and Multi-Physics Simulation

Another major challenge toward clinical translation is the limited capability of current predictive models to accurately simulate the time-dependent degradation and mechanical evolution of biodegradable Fe-based implants. Most corrosion models developed for BMs still rely on simplified assumptions such as uniform thickness reduction and decoupled mechanical loading, which neglect the complex chemo-mechanical interactions occurring during degradation. As highlighted in a comprehensive review by Joshi et al. [90], current computational approaches for BMs remain largely phenomenological, often calibrated to mass-loss data without explicitly accounting for microstructural heterogeneity or evolving stress fields. More advanced numerical frameworks have recently been proposed for Mg alloys, where phase-field modeling has been successfully used to simulate localized pitting corrosion and its interaction with mechanical stresses [91]. In that work, Kovacevic et al. [91] demonstrated that stress concentrations significantly accelerate localized dissolution, underscoring the necessity of coupling electrochemical kinetics with mechanical fields to obtain realistic lifetime predictions. Similarly, Saconi et al. [92] implemented a finite element model incorporating user-defined corrosion laws to predict strength loss in WE43 Mg alloy, showing that the stress redistribution induced by degradation strongly influences mechanical integrity over time.

Despite these important advances, such multi-physics modeling strategies have not yet been systematically extended to Fe-Mn-C systems. This limitation is particularly critical because Fe-Mn-C alloys exhibit phase-dependent electrochemistry (γ-austenite vs. ε-martensite), deformation mechanisms dependent on SFE, and stress redistribution caused by porosity, features that introduce additional complexity compared to Mg alloys. Furthermore, degradation product accumulation, which is known to significantly slow in vivo degradation of Fe-based implants, is rarely incorporated into numerical simulations. Consequently, current models cannot reliably predict the coupled evolution of microstructure, degradation morphology, mechanical performance, and surrounding bone response. The absence of validated multi-physics frameworks integrating electrochemical kinetics, microstructural evolution, mechanical loading, and biological remodeling therefore remains a major barrier to accurate implant lifetime estimation and safety margin definition for Fe-based biodegradable devices.

### 6.4. Insufficient Long-Term In Vivo Validation

Long-term in vivo validation remains a major translational restriction for biodegradable Fe-based alloys. Although in vitro cytocompatibility studies consistently demonstrate acceptable cell viability, often exceeding 80% for Fe-Mn systems [69,93,94], these short-term assays do not reproduce the complex physiological conditions that govern DR, ion transport, tissue remodeling, and mechanical loading in vivo. More recent animal studies have begun to address this gap, yet the available evidence remains limited in duration and scope. In this respect, Traverson et al. [82] evaluated Fe-30Mn alloy implants in a rat femoral model over six months and reported favorable biocompatibility with enhanced bone apposition compared to SS 316L controls. Importantly, no significant systemic toxicity was observed. However, degradation remained relatively slow, indicating that Mn alloying alone does not fully resolve the intrinsic low DR of Fe-Mn-based implants in vivo.

AM has further enabled investigation of porous Fe-Mn systems under physiological conditions. In a 48-week implantation study, Li et al. [95] assessed 3D-printed porous Fe-30Mn scaffolds fabricated by LPBF and demonstrated stable osseointegration and absence of pathological alterations in major organs. While porosity promoted tissue ingrowth and interfacial stability, complete material resorption was not achieved within the experimental timeframe, highlighting the persistent challenge of long-term degradation control. Similarly, Saliba et al. [96] investigated Fe-Mn and Fe-Mn-Ag pins in a rat model and reported antibacterial functionality and absence of adverse systemic effects. Nevertheless, substantial portions of the implants remained structurally intact, further confirming that in vivo DR remain slower than often predicted by immersion testing. Beyond rodent models, a large-scale animal study conducted by Wegener et al. [97] evaluated a porous Fe-based degradable bone substitute in a sheep model and reported localized inflammatory responses associated with degradation product deposition, emphasizing the importance of extended implantation time and higher-fidelity biomechanical environments when assessing translational feasibility.

**Figure 4:**
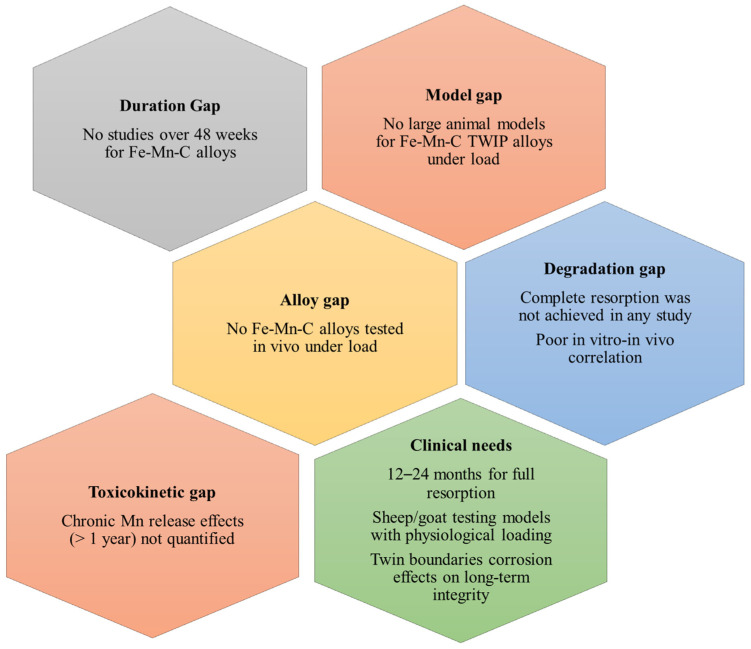
Critical gaps identified for the investigation of the in vivo degradation of Fe-Mn-C alloys for load-bearing applications.

Despite these advances, systematic in vivo investigations of Fe-Mn-C TWIP alloys under clinically relevant load-bearing orthopedic conditions remain largely absent. The presence of deformation twins and SFE-dependent plasticity introduces microstructural heterogeneities that may influence localized corrosion pathways and interfacial stability. Twin boundaries could act as preferential electrochemical sites, yet their contribution to long-term tissue integration or inflammatory response has not been experimentally clarified. Furthermore, while Mn accelerates degradation, the cumulative systemic effects of sustained Mn ion release during multi-year degradation remain insufficiently quantified. Although Mn is an essential trace element, chronic overexposure has been associated with neurotoxicity in non-implant contexts [98], highlighting the need for long-term toxicokinetic assessment specific to biodegradable orthopedic devices. Additionally, the synchronization between implant degradation and the staged process of bone healing, from inflammation and callus formation to mineralization and remodeling, has not yet been mechanistically established for Fe-Mn-C systems. Figure 4 summarizes the critical gaps facing the clinical translation of Fe-Mn-C-based alloys for biodegradable orthopedic implants.

### 6.5. Lack of Standardized Testing and Regulatory Frameworks

In parallel with biological validation challenges, the absence of standardized testing protocols specifically tailored to biodegradable Fe-based materials remains a significant regulatory obstacle. Current evaluation methodologies are largely adapted from permanent metallic implant standards or from Mg-based biodegradable systems, despite fundamental differences in degradation mechanisms and rates. Fe-based alloys degrade primarily through the formation of relatively stable oxide and phosphate degradation product layers that may partially passivate the surface and progressively slow dissolution, in contrast to the rapid anodic dissolution and hydrogen evolution characteristic of Mg-based alloys [9]. Consequently, there is no consensus regarding acceptable DR windows for Fe-based orthopedic implants, standardized immersion durations that realistically replicate physiological buffering and protein adsorption, or validated protocols for simultaneous mechanical loading and corrosion exposure. Moreover, clinically safe Mn ion release thresholds specific to biodegradable Fe-Mn systems have not been formally harmonized within regulatory frameworks. This lack of methodological standardization complicates cross-study comparison, increases variability in reported DRs, and delays regulatory approval processes. A structured comparison between currently applied testing methodologies and proposed Fe-specific standardization needs is summarized in Table 10.

In summary, although Fe-Mn-C-based alloys demonstrate promising mechanical compatibility, non-magnetic behavior, and alloy-tunable degradation characteristics, their clinical translation is constrained by limited long-term in vivo data, incomplete understanding of microstructure-driven degradation under load-bearing conditions, uncertainty regarding sustained Mn exposure, and absence of harmonized testing standards. Addressing these gaps through multi-year large-animal studies, corrosion-mechanics coupled evaluations, and standardized regulatory frameworks will be essential to bridge the transition from laboratory-scale innovation to safe orthopedic clinical application.

## 7. New Opportunities for Innovation

The translation of Fe-Mn-C biodegradable alloys from laboratory curiosities to clinically viable orthopedic fixation implants demands innovation beyond incremental improvements in alloy composition or processing parameters. The following sections critically examine five frontier areas including architectured porosity, hybrid systems, surface functionalization, smart degradation control, and AM personalization, each representing a distinct strategy to address the fundamental tension between mechanical integrity and controlled degradation that has constrained Fe-based biodegradable alloys for decades.

### 7.1. Architectured and Graded Porosity

**Figure 5:**
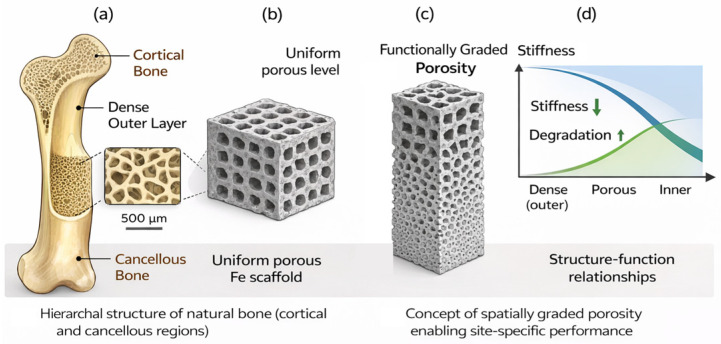
Evolution of architectural design in biodegradable Fe-based scaffolds. (**a**) hierarchical structure of natural bone; (**b**) additively manufactured uniform porous Fe scaffold; (**c**) concept of functionally graded porosity, and (**d**) the corresponding structure-function relationships.

The architectural design of implant porosity has evolved substantially from early studies that treated porosity only as a degradation accelerator toward sophisticated functionally graded architectures that spatially coordinate mechanical and biological performance. This evolution reflects a growing recognition that bone itself is not a uniform material but a hierarchically organized composite, with dense cortical bone at the periphery transitioning to porous cancellous bone in the interior, each region serving distinct mechanical and biological functions. Recent advances in AM [78,106,107,108] have finally enabled the fabrication of metallic implants that mimic this natural organization, creating opportunities to match implant stiffness and degradation profiles to the specific requirements of different anatomical sites. As illustrated in Figure 5, this design paradigm enables the development of functionally graded biodegradable scaffolds, where structural features are engineered to achieve site-specific performance and improved integration with the surrounding tissue.

The current state of the art in architectured porosity for Fe-Mn systems reveals both significant progress and persistent limitations. Salama et al. [78] systematically investigated topologically ordered porous Fe structures, demonstrating that DR can be tuned from 0.625 to 1.640 mmpy through control of strut thickness and unit cell geometry, with cubic and truncated octahedron configurations showing particularly promising combinations of permeability and mechanical stability. However, similar studies also reveal a critical constraint lies in the relationship between pore architecture and degradation mode that remains inadequately understood. While highly interconnected porous structures with porosity exceeding 80% undoubtedly accelerate degradation through increased surface area and enhanced electrolyte penetration, they simultaneously compromise fatigue resistance under the cyclic loading conditions characteristic of long bone fixation [74]. This creates a design paradox where the architectural features that enhance DRs may undermine the mechanical performances required for load-bearing applications.

Functionally graded porosity represents an emerging solution to this paradox, though its implementation remains empirically driven rather than mechanistically optimized. Liu et al. [69] successfully fabricated Fe-Mn scaffolds using sponge replication techniques that achieved 85% porosity with elastic moduli of 0.12–0.37 GPa, values approaching those of cancellous bone, and DRs of approximately 0.8 mmpy. Yet these achievements mask underlying scientific challenges that have not been adequately addressed in the literature. The interfaces between dense and porous regions create inevitable stress concentrations that may initiate crack propagation under physiological loading, while manufacturing defects at these transition zones introduce electrochemical heterogeneity that promotes localized rather than uniform degradation. Furthermore, the optimal grading profile for synchronized degradation and mechanical loss remains determined by trial and error rather than predictive modeling. Current designs typically employ linear or stepped porosity gradients, but there is no theoretical justification for these profiles over more complex non-linear architectures that might better match the spatially varying stress fields and bone regeneration rates in actual fractures. The integration of topology optimization algorithms with time-dependent corrosion models represents a critical research frontier. For instance, the work conducted by Mehboob et al. [109] on optimized bone plates remains limited to static mechanical considerations without incorporating the evolving material properties that characterize biodegradable implants.

### 7.2. Hybrid Metallic-Bioactive Systems

The concept of hybrid systems integrating BMs with bioactive ceramics has gained substantial traction as a strategy to simultaneously address multiple limitations of monolithic metallic implants. By combining the mechanical integrity of Fe-Mn matrices with the osteoconductive and degradation-accelerating properties of ceramic phases, particularly hydroxyapatite, researchers aim to create composite materials that exceed the performance of either constituent alone. This approach is grounded in solid mechanistic principles, as the introduction of ceramic particles creates microgalvanic coupling with the Fe matrix, increases interfacial defect density due to thermal expansion mismatch, and generates secondary phases that destabilize protective oxide layers, all of which contribute to accelerated degradation [110,111]. In this regard, Heiden et al. [68] provided an important evidence for this approach, demonstrating that porous Fe30Mn-10HA composites achieved DRs of 0.82 ± 0.04 mmpy, a forty-fold increase compared to nonporous Fe30Mn at 0.02 mmpy. Their work elucidated the specific mechanism of acceleration, identifying the formation of Ca_2_Mn_7_O_14_ phases at the Fe-HA interface as critical to destabilizing passive oxide layers and promoting active dissolution.

The optimization of ceramic content presents an additional materials science challenge that remains incompletely resolved. While low HA loadings (5–10 wt.%) provide bioactive benefits without severe mechanical compromise, higher concentrations (>15 wt.%) introduce brittleness that is fundamentally incompatible with the deformation requirements of orthopedic fixation devices. This phenomenon has been systematically documented by Dehestani et al. [112], who prepared Fe-HA composites through PM with varying HA compositions and particle sizes, demonstrating that when 2.5 wt.% HA was added, the YS and TS of all samples were reduced to half the value for pure Fe, with mechanical properties decreasing progressively as HA content increased.

The ductility that makes Fe-Mn-C alloys attractive for load-bearing applications derives from their capacity for extensive plastic deformation through twinning and dislocation motion, a capacity that ceramic particles disrupt by creating stress concentration sites and promoting crack initiation [113]. Furthermore, the long-term in vivo behavior of Fe-Mn-HA interfaces remains virtually unexplored, with no studies exceeding 12 months duration examining whether the accelerated degradation observed in vitro persists or whether biological encapsulation of the implant slows degradation over time, as observed in monolithic Fe implants [67]. This knowledge gap is particularly critical given that Heiden et al. [68] observed the formation of Ca_2_Mn_7_O_14_ phases at Fe-HA interfaces during sintering, which may behave unpredictably under long-term biological exposure despite accelerating initial DR.

**Figure 6:**
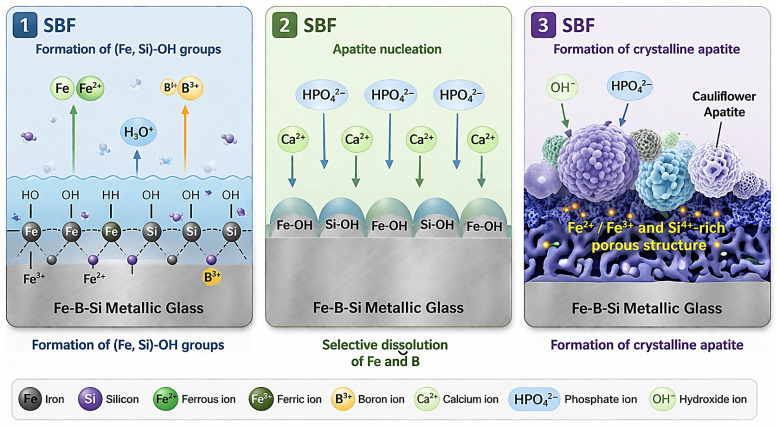
Schematic representation of the bioactivity mechanism of Fe-B-Si metallic glass in simulated body fluid (SBF), highlighting the formation of surface (Fe, Si)-OH groups, selective dissolution, subsequent apatite nucleation, and the growth of a crystalline apatite layer. Adapted from Qin et al. (2016) [114].

An alternative to Fe-HA composites is the development of intrinsically bioactive Fe-based alloys in which surface chemistry itself promotes mineralization. In this context, Qiu et al. [114] demonstrated that Nb-containing Fe-based metallic glasses spontaneously form apatite in SBF without surface treatment. Octacalcium phosphate nucleated within 1 day, followed by the development of a bone-like hydroxyapatite layer after 3 days. This process (shown in Figure 6) was driven by selective dissolution of Fe and B, and rapid formation of Si-OH, Fe-OH, and Nb-OH functional groups that promoted Ca^2+^ and phosphate adsorption. Unlike particulate Fe-HA systems, bioactivity here arises from controlled surface reactions and hierarchical porosity formation, highlighting that hybrid functionality can be achieved through compositional design rather than external ceramic reinforcement.

Beyond hydroxyapatite, bioactive glass coatings represent an alternative hybrid strategy with distinct advantages and limitations. These silicate-based materials enhance osseointegration through rapid surface apatite formation and provide controlled ion release (Si, Ca, P) that may stimulate osteogenesis. The bioactive mechanism involves the formation of a silica-rich layer at the interface with body fluids by the migration of ions from the glass, which promotes the nucleation of hydroxycarbonate apatite and creates a chemical bond with surrounding bone tissue [115]. Specifically, bioactive glasses release Ca, P, and Si ions that promote the production of hydroxyapatite, a valuable component of bone that enhances the healing process [115,116]. However, the application of bioactive glass to Fe-Mn substrates faces a critical materials compatibility challenge, as the thermal expansion coefficient (CTE) mismatch between silicate glasses and Fe often causes coating delamination during processing or early implantation, compromising both the protective and bioactive functions of the coating. The CTE of standard 45S5 bioactive glass is approximately 15.1 × 10^−6^ K^−1^, while Fe-based alloys have CTE values of ~10–30 × 10^−6^ K^−1^, creating significant thermal residual stresses during sintering that may result in cracking or delamination [117,118]. Furthermore, laser cladding techniques used for bioactive glass deposition operate at temperatures of 1000–1500 °C, where post-treatment cracking occurs due to the significant difference in CTE between the bioactive glass and the substrate material [118].

### 7.3. Surface Functionalization Strategies

Surface functionalization offers a unique multipurpose approach to biodegradable implant design because it enables temporary decoupling of the initial biological response from long-term degradation behavior. Unlike bulk alloying, which permanently fixes material properties, surface modifications can provide intense but time-limited effects, protecting the implant during the critical early healing phase when bone integration is established, then permitting or even accelerating degradation once mechanical continuity with the host tissue has been achieved. This temporal control is particularly valuable for Fe-Mn-C alloys, where the intrinsic DR is often too slow for clinical requirements but permanent acceleration would compromise early mechanical stability [119,120].

Laser surface texturing provides an alternative functionalization pathway with distinct characteristics. Femtosecond and nanosecond laser ablation creates super-hydrophilic surfaces with nanostructured oxide layers that significantly enhance DRs through increased surface area and the formation of metastable oxide phases [121,122]. In this regard, Donik et al. [123] demonstrated that laser-textured Fe-Mn surfaces exhibit CRs eight-fold to thirteen-fold higher than polished counterparts, attributing this acceleration to nanoscale surface features and elevated Mn content in the modified oxide layer. Specifically, their X-ray photoelectron spectroscopy analysis revealed that laser texturing transforms the surface chemistry, converting the majority of Fe into corrosion-favorable Fe_2_O_3_ (up to 60 at% of the oxide layer) with significantly elevated Mn content compared to the bulk alloy. However, this approach introduces critical concerns that limit its clinical viability. The long-term stability of laser-induced nanostructures under the mechanical loading and fretting conditions characteristic of orthopedic implants remains unverified. Here, early surface modifications may be deteriorated or mechanically disrupted, leading to unpredictable transitions in degradation behavior. Additionally, the increased surface area that accelerates degradation may simultaneously enhance hydrogen evolution or metallic ion release to levels that compromise biocompatibility. These effects have not been adequately characterized for textured Fe surfaces. Recent work by Sun et al. [124] confirmed that femtosecond laser-induced nano-ripple structures on Fe-30Mn surfaces increase the actual biodegradation rate while maintaining cytocompatibility, though the study was limited to 30-day in vitro observations.

The frontier of surface functionalization lies in smart, responsive coatings that adapt their properties to physiological signs. Self-healing coatings based on pH-responsive nanocontainers, such as halloysite nanotubes, mesoporous silica nanoparticles, or layered double hydroxide shells loaded with corrosion inhibitors or bioactive agents, can autonomously repair mechanical damage and modulate ion release in response to local environmental changes. In this respect, Sanyal et al. [125] provide a comprehensive review of these technologies, noting that micro/nanocontainers typically range from 50–200 µm for micro-scale containers and smaller for nanocontainers, designed to respond to external environmental stimuli through intelligent, timely release of encapsulated agents. Specifically, pH-responsive coatings exploit the pH changes that occur during degradation, typically acidic at anodic sites and alkaline at cathodic sites, to trigger inhibitor release. Recent work carried out by Ouyang et al. [126] demonstrated mesoporous silica core layered double hydroxide shell nanocontainers (MSN-MBT@LDH) that respond to acidic environments by releasing 2-mercaptobenzothiazole corrosion inhibitors, achieving robust corrosion protection with significantly enhanced barrier properties compared to control coatings. Similarly, Dehghan et al. [127] developed chitosan-ImH@γ-cyclodextrin metal-organic framework composites that exhibit 97.27% inhibition efficiency after 5 days in SBF by releasing imidazole in response to pH increases at corrosion sites.

While extensively studied for Mg-based alloys, where acidic conditions accelerate corrosion, adaptation to Fe-Mn systems requires careful consideration of the fundamentally different electrochemistry of Fe. Unlike Mg, whose corrosion produces soluble Mg^2+^ and hydroxide ions, Fe forms insoluble oxide layers that may behave differently in response to pH variations, potentially slowing rather than accelerating degradation under acidic conditions [128]. This inverse response to environmental stimuli complicates the direct transfer of smart coating concepts from Mg to Fe systems and necessitates fundamental research into stimuli-responsive mechanisms specific to Fe biodegradation. Furthermore, as noted by Rabeeh and Hanas [67] in their review of Fe-based biodegradable implants, the long-term in vivo stability of surface modifications, including smart coatings, remains inadequately validated, with particular concern regarding coating delamination, mechanical wear, and the biological response to released encapsulation materials. The development of triple-stimuli-responsive nanocontainers that respond to acid, alkali, and corrosion potential simultaneously, as demonstrated by Wang et al. [129] for Al alloys, may offer a pathway forward for Fe systems where single-stimulus pH responsiveness is insufficient.

### 7.4. Smart Degradation Concepts

The concept of smart biodegradable implants capable of autonomously modulating their DRs in response to the biological environment represents the most ambitious frontier in orthopedic biomaterials research. These systems aim to achieve what current biodegradable metals cannot, which is the perfect synchronization between implant degradation and the staged biological process of bone healing, from initial inflammation through soft callus formation, mineralization, and ultimate remodeling. While this vision remains largely unrealized for Fe-based systems, recent advances in materials science and bioelectronics have established conceptual foundations that may eventually enable its implementation. The convergence of smart and biodegradable polymer technologies is about to transform biomedical engineering, particularly in regenerative medicine and minimally invasive devices, though few smart biodegradable systems have reached clinical use despite strong laboratory data [130].

Current research explores multiple stimuli-responsive strategies, each with distinct mechanisms and limitations. pH-responsive degradation represents the most intuitively appealing approach, given that the inflammatory phase following implantation creates localized acidic environments with pH values of 5.0–6.5 due to macrophage and osteoclast activity [131]. Coatings designed to accelerate dissolution under these acidic conditions could theoretically match implant DRs to initial bone formation, then stabilize as pH normalizes during healing. Recent work on Mg alloys has demonstrated this principle using mPEG-PLGA coatings that selectively dissolve in alkaline environments (pH ≥ 10) created by Mg degradation, effectively buffering pH and modulating Mg^2+^ release [132]. However, this approach faces a fundamental materials science challenge when applied to Fe. Unlike Mg, whose corrosion is accelerated by acid through rapid hydrogen evolution and chloride penetration, Fe corrosion typically slows under weakly acidic conditions due to oxide layer destabilization followed by repassivation. This inverse response to pH complicates the design of responsive coatings and may require alternative triggering mechanisms based on specific inflammatory mediators rather than pH alone. The Pourbaix diagram for the Mo-H_2_O system reveals that Mo species stability varies significantly with pH, suggesting that environment-adaptive alloys may offer alternative pathways for stimuli-responsive degradation [133].

Mechano-responsive systems that adjust degradation based on load transfer to healing bone offer an alternative paradigm with strong physiological rationale. The concept involves implants that accelerate degradation once mechanical continuity with the host skeleton is established, detected through integrated piezoelectric sensors or strain-responsive polymers [134]. Shape-memory wires manufactured from Ni-Ti alloys have been integrated into orthopedic implants to stimulate bone repair through controlled micromovements, with the capability to exert substantial tensile force and function as sensors that make the healing process visible through electrical resistance measurements [135]. However, Ni-Ti alloys are not biodegradable, and adaptation to Fe-Mn alloys faces several integration challenges. Biodegradable sensors compatible with Fe-Mn alloys, wireless power transfer systems, and biocompatible actuation mechanisms must all be developed and validated, representing a highly interdisciplinary initiative covering materials science, electrical engineering, and orthopedic surgery. Current technology readiness levels for such systems remain at early conceptual stages, with no demonstration of closed-loop degradation control in any animal model.

Electrochemical modulation through external electromagnetic fields provides a less invasive but potentially less accurate alternative. For example, recent work on Mo-based biodegradable metals has demonstrated that electrochemical behavior varies significantly with inflammatory mediators such as hydrogen peroxide and albumin, suggesting opportunities for environment-adaptive alloys even without complex sensor integration. In this regard, Gao et al. [136] compared the long-term corrosion profile of Mo with Mg, Zn, and Fe, demonstrating that Mo exhibited the lowest DR with the most uniform degradation mode, and importantly, performed bilaterally under simulated inflammatory conditions with hydrogen peroxide and bovine serum albumin. However, clinical translation of electrochemical modulation would require patients to wear external devices or undergo periodic clinical procedures, raising compliance and practicality concerns that have not been addressed in the literature. Furthermore, the substantial difference in DRs between Mo (0.0336 mmpy in vitro) and Fe (0.10 mmpy) complicates the direct transfer of electrochemical modulation strategies from one metal system to another [137].

The critical scientific gap across all smart degradation concepts is the absence of demonstrated closed-loop control, systems that autonomously adjust DRs based on real-time feedback from bone healing biomarkers. Achieving this capability requires integration of biodegradable sensing elements, reliable wireless communication, and biocompatible actuation mechanisms within a single implantable device, then maintaining this functionality throughout the months-long degradation process. Current wireless biodegradable sensors employ LC resonators with inductive coils and capacitive elements fabricated from degradable metals such as Mg or Zn, paired with biodegradable dielectric layers [138]. These systems have demonstrated wireless readout at distances up to 5 mm ex vivo and around 2 mm in vivo for intracranial pressure monitoring, with the quality factors (Q) gradually decreasing as implants partially degrade [138]. However, the translation of these proof-of-concept devices to closed-loop degradation control for orthopedic fixation requires solving fundamental challenges. One of these challenges is the development of sensors that respond to bone healing biomarkers (e.g., specific cytokines, mineralization markers) rather than simply measuring physical parameters. Second, creating actuation mechanisms that can modulate Fe-Mn-C DRs in response to sensor feedback without external power, and finally, ensuring that the integrated system maintains mechanical integrity sufficient for load-bearing applications while degrading at controlled rates. This represents perhaps the most significant challenge in biodegradable metals research, requiring convergence of expertise rarely found within single research groups or even single institutions. As noted in recent reviews, despite strong laboratory data, few smart biodegradable polymers or metals have reached clinical use, and regulatory strategies need to connect with the dynamic behavior of these materials, which challenge existing medical device classification systems [130].

### 7.5. Additive Manufacturing and Implant Personalization

Additive manufacturing has transitioned from a prototyping curiosity to a clinically implemented technology for patient-specific orthopedic implants, with the global market for 3D-printed orthopedic devices exceeding $2 billion in 2022 and projected growth exceeding 11% annually through the next decade [139]. For Fe-Mn-C biodegradable alloys, LPBF offers unique capabilities that extend beyond simple shape customization to fundamental control over microstructure, degradation behavior, and mechanical properties. The rapid solidification characteristic of LPBF creates fine grain structures and metastable phase distributions that accelerate degradation compared to conventionally processed materials, while precise control over local laser parameters enables spatial variation of porosity and microstructure within single implants [76].

However, the manufacturing of biodegradable Fe-based alloys via LPBF faces critical challenges that have not been adequately resolved in the current literature. For instance, microstructural heterogeneity induced by thermal cycling during layer-by-layer fabrication creates varying distributions of γ-austenite and ε-martensite depending on local scanning parameters, leading to spatially variable corrosion behavior that may compromise implant reliability. In this regard, Liu et al. [71] demonstrated that scanning speeds between 600 and 900 mm/s produce corrosion rates ranging from 0.09 to 0.22 mmpy depending on phase distribution, yet the reproducibility of these relationships across different LPBF systems, powder batches, and build orientations remains inadequately characterized. This variability poses significant challenges for regulatory approval, where consistent, predictable performance must be demonstrated across manufacturing lots [140].

Selective evaporation of Mn during high-energy laser processing presents an additional compositional challenge with metallurgical consequences. The boiling point of Mn (2061 °C) is considerably lower than that of Fe (2862 °C), creating preferential evaporation from the melt pool that may alter designed Mn content, and consequently the SFE and phase stability, in surface regions where degradation initiates. Recent work carried out by Gärtner et al. [141] on Fe-Mn-Si shape memory alloys processed by LPBF demonstrated pronounced Mn evaporation that affects the primary solidification phase and phase fractions in the final microstructure, with Mn content decreasing by approximately 0.3% during the production process compared to the raw powder feedstock. This effect is particularly pronounced at high laser power densities and may result in gradients of composition and properties through the implant thickness that are not present in the original powder feedstock. In this respect, Ewald et al. [142] observed even more substantial Mn losses of around 3% in Fe-Mn-Al-Ni alloys during LPBF processing, attributed to the higher vapor pressure of Mn compared to other alloying elements. Post-process heat treatments to homogenize composition risk coarsening the fine grain structures that provide beneficial mechanical properties, creating a processing optimization dilemma that has not been systematically addressed.

The surface finish of as-printed Fe-Mn-based alloys presents a final manufacturing consideration with competing effects on performance. LPBF produces surfaces with roughness values typically exceeding 10 μm Ra, which accelerates initial corrosion through increased surface area and electrolyte trapping but may compromise fatigue performance by creating stress concentration sites [28]. Post-processing through mechanical polishing, chemical etching, or heat treatment modifies surface chemistry and must be optimized specifically for biodegradable metals. The removal of surface material during polishing may eliminate the very microstructural features (fine grains, metastable phases) that make LPBF attractive for biodegradable implants application, while chemical treatments may introduce contamination or alter surface oxide chemistry in ways that unpredictably affect degradation.

The future of personalized biodegradable implants lies in the integration of digital twin technology, which consists of virtual representations that combine patient-specific anatomical models, biomechanical loading simulations, and predictive degradation algorithms to optimize implant design before fabrication [143]. In this regard, Andres et al. [140] developed a clinically applicable digital twin workflow that integrates patient-specific imaging, motion capture, musculoskeletal modeling, and finite element simulation for orthopedic trauma surgery, demonstrating feasibility across five real patient cases involving different anatomical sites and treatment strategies. Their work demonstrated that digital twins can predict improvements in implant stress distribution and fracture strain states, offering valuable insights for optimizing surgical decisions. For Fe-Mn-C systems, comprehensive digital twins must couple finite element mechanical analysis with electrochemical corrosion modeling, bone remodeling simulations, and machine learning-based prediction of patient-specific healing rates. This integration represents a significant computational challenge, as the coupled physics of mechanical loading, electrochemical dissolution, and biological remodeling operate on vastly different time scales and length scales that current simulation frameworks struggle to bridge [143,144].

Regulatory and standardization barriers present perhaps the most immediate obstacle to clinical translation of personalized biodegradable implants. Current FDA and EU MDR pathways were developed for either standard, mass-produced devices or patient-specific bioinert implants, with no established framework for personalized biodegradable devices [145]. As noted in recent comprehensive reviews, 3D-printed medical devices tailored specifically to a patient’s anatomy are custom-made and hence fall under regulations for personalized devices, but the need for specific regulations covering patient-specific printed devices outside of emergency or compassionate cases remains unmet [139]. In the United States, patient-specific 3D-printed implants fall under FDA’s 510(k) pathway, which requires demonstration of significant equivalence to legally marketed predicate devices, but the manufacturing processes must be validated per the Quality System Regulation (21 CFR 820) to ensure requirements are continually met [146]. The FDA has not created an autonomous additive manufacturing-specific regulatory framework. Instead, its primary direction is provided through preliminary 2017 guidance that is tentative and subject to change [146]. In the European Union, orthopedic implants are regarded as Class III medical devices associated with the highest risk, requiring extensive clinical testing with reported timelines of 3–7 years [147]. While custom-made devices can be approved under Annex XIII of the Medical Device Regulation with submission of a technical note describing design, manufacturing, testing, and risk assessment, all cases to date have been approved as exceptional or compassionate cases rather than through standard regulatory processes [139]. Each patient-specific implant currently requires individual validation, creating regulatory barriers that prevent large-scale clinical implementation. The development of modular validation frameworks, where patient-specific variations remain within pre-validated design spaces defined by computational modeling and mechanical testing, represents a critical need that requires collaboration between regulatory scientists, computational modelers, and orthopedic surgeons to establish acceptable evidence standards for safety and efficacy.

### 7.6. Manufacturing Scalability and Industrial Compatibility

A critical but underexplored dimension of clinical translation for Fe-Mn-C biodegradable alloys is their practical manufacturability within the existing orthopedic implant industry. From a raw material cost perspective, Fe-Mn-C alloys offer a structural advantage over competing biodegradable metallic systems. In this regard, Fe and Mn are globally abundant, relatively low-cost commodity metals compared to the Ti and Co-Cr alloys currently dominating the implant market [139]. At the bulk alloy level, the Fe-Mn-C compositions required for TWIP behavior (Fe; 15–25 wt.% Mn; 0.3–0.8 wt.% C) can be produced via electric arc furnace melting and conventional continuous casting routes that are already established in the specialty steel and biomedical manufacturing industries, representing a significant scalability advantage over Mg-rare-earth systems, whose production requires specialized reduction metallurgy and strict atmospheric control.

However, the shift toward LPBF-based manufacturing introduces higher manufacturing costs. Gas-atomized Fe-Mn powder feedstock suitable for LPBF typically requires significant investment per kilogram, and machine time, post-processing, and quality inspection adding further expense [139]. Current LPBF build volumes limit throughput to a small number of implants per batch, making unit costs higher than conventionally machined implants. Cost modeling for patient-specific LPBF-fabricated implants suggests manufacturing costs 3–8 times higher than conventionally machined equivalents [139].

Compatibility with existing orthopedic manufacturing infrastructure is an important strategic consideration. Standard fixation devices including bone plates, cortical screws, and intramedullary nails, are conventionally produced by Computer Numerical Control (CNC) machining from bar or billet stock [139]. Fe-Mn-C alloys can, in principle, be processed by this route from cast and thermomechanical processed billets, leveraging existing machining infrastructure. Their machinability is broadly comparable to austenitic stainless steels, which are extensively machined for orthopedic applications [147]. Surface finishing, including passivation, electropolishing, and sterilization by ethylene oxide or gamma irradiation, would also be compatible with established orthopedic manufacturing workflows.

In summary, Fe-Mn-C alloys can be used in large-scale orthopedic manufacturing, but the production method should be chosen based on the application. Conventional machining is suitable for simple, standard implants in the short term, while LPBF is better for complex, patient-specific designs as costs decrease and regulations evolve.

The innovation strategies discussed in Section 7 cover a wide range of technological maturity, which directly shapes their realistic path to clinical use. Table 11 provides an estimated Technology Readiness Level (TRL) for each strategy, based on available experimental evidence, validation stage, and proximity to implementation. Beyond ranking maturity, the table identifies the specific gaps that must be closed to advance each approach, from the relatively near-term potential of AM-enabled personalization to the early stage promise of smart degradation control, offering a practical roadmap for prioritizing future research in Fe-Mn-C biodegradable systems.

## 8. Conclusions

This review has critically examined the development of Fe-Mn-C biodegradable alloys as next-generation orthopedic fixation devices, tracing the evolution from fundamental materials design through processing innovations to the challenges currently impeding clinical translation. Fe-Mn-C alloys have emerged as the most promising candidates among Fe-based alloys for load-bearing applications, offering a unique combination of mechanical strength (400–900 MPa), exceptional ductility (>50% elongation through TWIP mechanisms), and tunable DRs. The integration of C as an austenite stabilizer and SFE modulator enables precise control over deformation mechanisms, while Mn additions reduce magnetic susceptibility for MRI compatibility and accelerate degradation compared to pure Fe. However, the fundamental tension between mechanical integrity and controlled degradation remains the central unresolved challenge, as the dense alloys degrade too slowly (~0.1–0.2 mmpy) for clinical relevance, while highly porous structures achieving acceptable rates (~0.8–1.0 mmpy) compromise fatigue resistance and structural reliability.

Processing innovations, particularly LPBF, have demonstrated remarkable capabilities for engineering porosity, microstructure, and degradation behavior simultaneously. Yet critical reproducibility challenges persist, such as the selective Mn evaporation during laser processing that alters designed compositions. Moreover, microstructural heterogeneity creates spatially variable corrosion behavior, and the absence of standardized protocols complicates cross-study comparison and regulatory approval. Most significantly, the persistent discrepancy between in vitro DRs and in vivo performance, where biological encapsulation and degradation product accumulation retard degradation, represents the primary translational barrier.

Several fundamental scientific questions demand attention. The coupling between microstructural features enhancing mechanical performance (twin boundaries, phase interfaces) and those promoting localized corrosion is inadequately understood. Current degradation models cannot predict time-dependent mechanical integrity loss under coupled chemo-mechanical conditions. Long-term in vivo validation of Fe-Mn-C TWIP alloys under load-bearing conditions is virtually absent, with no studies exceeding twelve months. The biological response to sustained Mn ion release, particularly regarding potential neurotoxicity, requires systematic toxicokinetic assessment.

Looking forward, clinical translation demands an integrated, multidisciplinary approach. Corrosion-mechanics coupled characterization must become standard practice, incorporating simultaneous mechanical testing with real-time corrosion monitoring. Closed-loop smart degradation control, integrating biodegradable sensors, wireless communication, and stimuli-responsive coatings, represents the most promising frontier for achieving true synchronization between implant degradation and bone healing, though current technology readiness levels remain low. Modular regulatory frameworks must be established with agencies to overcome the current requirement for individual device validation that prevents scalable implementation. Large-animal studies with minimum two to three-year duration are essential to validate long-term performance under relevant biomechanical conditions. Digital twin integration offers revolutionary potential for personalized implant design, though realizing this requires multi-physics simulation frameworks capable of bridging the vastly different time scales of mechanical loading, electrochemical dissolution, and biological remodeling.

Fe-Mn-C biodegradable alloys stand at a critical juncture. The materials science foundations are established, processing capabilities are available, and clinical need is pressing. Success now depends on addressing the complex, interdisciplinary challenges separating promising laboratory results from reliable clinical performance. The development of truly smart, patient-specific, biodegradable orthopedic implants represents one of the most significant opportunities in modern biomaterials science, but achieving this vision will require sustained, collaborative effort transcending traditional disciplinary boundaries among materials engineers, bioelectronics specialists, computational modelers, regulatory scientists, and orthopedic surgeons.

## Figures and Tables

**Table 1:** Summary of conventional metallic biomaterials used for orthopedic bone plate applications [4,5,6,7].

Alloy	Elastic Modulus (GPa)	Yield Strength (MPa)	Merits	Limitations	Orthopedic Applications
316L stainless steel	~190	~200	Low cost, good mechanical strength, corrosion resistance, good toughness	High stiffness (stress shielding), Ni ion release, moderate biocompatibility	Rods, nails, plates, screws, pins, wires, cables
**Ti and Ti-based alloys**	55–100	530–900	Lightweight, corrosion resistant, better biocompatibility	Elastic modulus mismatch, potential release of toxic alloying elements (e.g., V, Al)	Plates, rods, screws, hip and knee prostheses
Co-Cr alloys	220–230	275–1585	High wear resistance, good corrosion behavior, long-term durability	Very high stiffness, poor machinability, Ni ion release, biological reactivity	Load-bearing joint replacements, prosthetic stems, plates, wires

**Table 4:** Biological roles, acceptable concentration ranges, and potential toxicological effects of Mg^2+^, Zn^2+^, and Fe^2+^/Fe^3+^ ions.

Ion	Major Biological Roles	Physiological Range (Serum) **	Biological Importance	Potential Toxicological Effects If Exceeded	Ref.
**Mg^2+^**	Enzyme cofactor (ATP metabolism, DNA synthesis), neuromuscular function, and bone mineralization	~0.7–1.1 mmol/L (1.7–2.5 mg/dL)	Essential for >300 enzymatic processes	Hypermagnesemia: cardiac arrhythmia, hypotension; local tissue irritation at high local release	[12,26]
**Zn^2+^**	Protein synthesis, immune function, bone formation, and antioxidant defense	~10–18 µmol/L (65–110 µg/dL)	Cofactor for >200 enzymes and supports osteoblast activity	Zn toxicity: nausea, vomiting, immune suppression at high systemic levels; possible cytotoxicity at high local concentrations	[11,30]
**Fe^2+/^Fe^3+^**	Hemoglobin/myoglobin synthesis, electron transport, and DNA synthesis	~12–30 µmol/L (60–170 µg/dL) *	Critical for oxygen transport and cellular respiration	Fe overload: oxidative stress and local inflammation. Potential promotion of reactive oxygen species (ROS) and fibrosis with persistent degradation products	[13,31]

* Serum iron values vary with gender, age, and testing method. ** Physiological reference ranges are based on healthy adult human data.

**Table 5:** Comparative overview of Mg-, Zn-, and Fe-based biodegradable metallic systems for orthopedic fixation [50,51,52,53,54,55].

Parameter	Mg-Based Alloys	Zn-Based Alloys	Fe-Based Alloys
E (GPa)	41–45	90–110	~210 (bulk)
YS (MPa)	65–250	20–300	170–900
DR (mmpy)	0.5–4	0.02–0.3	0.05–0.2 (bulk)
Hydrogen evolution	Yes	No	No
Mechanical suitability	Limited for high load	Moderate load	Excellent for load-bearing
Main limitation	Rapid degradation	Limited ductility	Slow degradation
Acceleration strategy	Coatings, alloying	Alloying, microstructure	Alloying, porosity, galvanic effects
Clinical approval	Yes (e.g., MAGNEZIX^®^)	Limited	No widespread approval

**Table 6:** Effect of porosity on elastic modulus and DR in porous Fe-based biodegradable alloys.

Alloy/System	Processing Method	Porosity (%)	Elastic Modulus (GPa)	Compressive Strength (MPa)	DR (mmpy)	Ref.
Fe-30Mn	Sponge replication + sintering	~85	0.12–0.37	6–10	~0.8	[69]
Fe-Mn-HA composite	Salt-leaching + sintering	~60–70	~1–5	20–50	0.02 (dense) → 0.82 (porous)	[68]
Porous Fe-Mn alloys	Space-holder/PM routes	42–76	5–25 (decreasing with porosity)	50–250 (porosity dependent)	Up to 2.71	[67]
Dense Fe-Mn (comparison)	Conventional sintering	<5	~180–210	>400	0.02–0.05	[68]

**Table 7:** Structure–processing–degradation coupling in powder metallurgical Fe-Mn biodegradable alloys.

Structural Feature	Origin in Processing	Mechanical Effect	Degradation Effect	Ref.
Low porosity (<10%)	High densification during sintering	High modulus (~200 GPa)	Slow corrosion (~0.1–0.2 mmpy)	[22]
Moderate porosity (~30–50%)	Controlled space-holder PM	Reduced modulus (80–150 GPa)	Increased corrosion (~0.4–0.8 mmpy)	[68]
High interconnected porosity (>70%)	Sponge/replica techniques	Modulus approaching cancellous bone	Accelerated corrosion (~1 mmpy)	[54,68]
Fe-Mn phase stabilization	Mn homogenization during sintering	Improved ductility	Higher corrosion than pure Fe	[70]

**Table 8:** LPBF processing-structure-degradation correlation in Fe-Mn and Fe-Mn-C alloys.

LPBF Processing Feature	Structural Outcome	Implication for Corrosion	Ref.
Scanning speed variation (600–900 mm/s)	Varying porosity and phase fraction (ε/α′)	Corrosion rate tuned (~0.09–0.22 mmpy)	[71]
Rapid solidification	Fine grains & mixed martensitic phases	High grain boundary activity accelerates corrosion	[71,72]
High ε-martensite fraction	Microgalvanic coupling with γ-phase	Enhanced local corrosion	[72]
Porosity and LPBF defects	Surface area and microcracks	Localized corrosion initiation	[28]

**Table 9:** Comparison of in vitro and in vivo degradation of Fe-based implants.

Material	Processing	In Vitro DR (mmpy)	In Vitro Medium	In Vivo Rate/Observation	In Vivo Site	Duration	Study
Fe-30Mn	PM	0.44–1.26	Modified Hanks’	Partial resorption; bone cell integration	Rat femur	6 months	[82]
Fe-30Mn	Casting, cold drawn	0.54	Osteogenic media	Mild inflammatory reaction; necrotic bone at interface			
Fe-(0.5–6.9)Mn	Casting, forged	0.2–0.5 (estimated)	-	No significant degradation (phosphate passivation film)	Mouse subcutis	9 months	[81]
Fe-10Mn-1Pd	Casting, heat treated	0.9 (immersion)	SBF	No local toxicity; clinical anomalies	Rat femur	52 weeks	[77]
Fe-21Mn-0.7C-1Pd		0.21					
Fe-HA (5%)	PM	0.24–1.03	SBF	Good tissue integration, low inflammatory response	Sheep radial bone	10 weeks	[83,84]
Fe-TCP (5%)		~0.5–0.6 (estimated)					
Fe-BCP (5%)		~0.4–0.5 (estimated)					
FeMnSiCa	Casting, annealed	0.80	Ringer solution	Improved osteoinduction, extensive surface corrosion	Rabbit tibia	28 days	[85]

**Table 10:** Current testing approaches for biodegradable Fe-based alloys and proposed standardization needs, including immersion conditions, corrosion-mechanics coupling protocols, ion-release assessment, and minimum in vivo validation duration.

Evaluation Category	Current Practice (Adapted Standards/Methods)	Limitations for Fe-Based Alloys	Proposed Fe-Specific Standardization Needs	Ref.
Static immersion corrosion testing	Immersion in SBF or PBS at 37 °C; mass loss and pH monitoring; adapted from ISO 10993-15 [99] and ASTM G31 [100]	Short durations (7–28 days); static media; no protein supply; overestimation of DR compared to in vivo due to absence of diffusion barriers	Long-term immersion (≥6 months); dynamic flow systems; inclusion of proteins (albumin); controlled buffering capacity	[99,101]
Electrochemical testing (OCP, PDP, EIS)	Potentiodynamic polarization and impedance in SBF; scan rates 0.1–1 mV/s	Short-term assessment; surface preparation strongly influences results; poor correlation with long-term DR	Standardized scan rates for Fe systems; combined electrochemical-long-term immersion protocols	[102]
Degradation rate reporting	mmpy from mass loss or Tafel extrapolation	Wide variability in calculation methods; inconsistent surface area correction; no consensus acceptable DR window	Harmonized DR calculation protocol; definition of target DR window (e.g., 0.2–0.5 mmpy for load-bearing Fe implants)	[13,50]
Mechanical testing under corrosion	Separate mechanical (ASTM E8 [103]) and corrosion tests	No simultaneous corrosion-mechanics coupling; no standardized slow strain rate corrosion testing for Fe biomaterials	Development of corrosion-fatigue and slow strain rate testing standards in physiological media	[90]
Ion release & cytotoxicity	ISO 10993-5 [104] cytotoxicity; ICP-MS ion quantification; extraction ratios 0.2 g/mL	No Mn-specific long-term systemic exposure limits defined for biodegradable implants; short extraction durations (24–72 h)	Long-term cumulative Mn release evaluation; toxicokinetic modeling; harmonized extraction duration	[98,105]
In vivo validation	Small animal (rat) models; 4–48 weeks; histology & μCT	Limited large-animal load-bearing models; no minimum implantation duration defined	Minimum 12–24-month load-bearing large-animal studies; standardized organ histopathology panels	[95,97]
Comparison to Mg Standards	Adaptation of Mg corrosion methodologies (hydrogen evolution, rapid mass loss)	Fe does not produce hydrogen gas significantly; passivation slows DR; different electrochemical mechanism	Fe-specific corrosion modeling incorporating oxide layer growth kinetics	[13,50]

**Table 11:** Estimated Technology Readiness Levels (TRL) for innovation strategies discussed in Section 7.

Innovation Area	TRL (Estimated)	Current Status	Key Gap Before Next TRL
Architectured & graded porosity	4–5	In vitro validation of DR and mechanical tuning via controlled porosity	In vivo fatigue performance and corrosion-mechanical coupling under cyclic loading
Hybrid metallic–bioactive systems	3–4	Proof-of-concept composites with improved DR and bioactivity; limited in vivo data	Long-term in vivo validation and interface stability under load
Surface functionalization	4–5	Accelerated DR via laser texturing; smart coating concepts validated in Mg systems	Adaptation to Fe systems and long-term mechanical durability
Smart degradation control	2–3	Conceptual stage; individual components demonstrated separately	Integration into functional implant and in vivo closed-loop validation
AM personalization geometry	5–6	LPBF-based patient-specific geometries demonstrated; workflow partially established	Process reproducibility, Mn control, and regulatory standardization

## Data Availability

No new data were created or analyzed in this study. Data sharing is not applicable to this article.
